# Exploring the option of student-run free health clinics to support people living with type 2 diabetes mellitus: a scoping review

**DOI:** 10.3389/fpubh.2023.1128617

**Published:** 2023-07-18

**Authors:** Kesava Kovanur Sampath, Yan Ann-Rong, Marrin Haggie, Timi Tapara, Sharon Brownie

**Affiliations:** ^1^Waikato Institute of Technology – Te Pukenga, Hamilton, New Zealand; ^2^University of Canberra, Canberra, ACT, Australia; ^3^Tu Tonu Rehabilitation Ltd., Hamilton, New Zealand; ^4^Swinburne University, Melbourne, VIC, Australia; ^5^Griffith University, Gold Coast, QLD, Australia

**Keywords:** type II diabetes, T2DM, student clinics, student run free clinics, cultural sensitivity, scoping review

## Abstract

Diabetes is a major cause of morbidity and premature mortality worldwide and now identified as a ‘public health emergency’ and a ‘modern and preventable pandemic’. Indigenous populations are disproportionately affected by type 2 diabetes mellitus (T2DM) and associated complications. Student run free clinics (SRFCs) may play an important role in the prevention and management of T2DM. The primary objective of this scoping review was to investigate the opportunity for curriculum enhancement through the role and effectiveness of SRFCs in managing T2DM. Electronic databases such as PubMed, CINAHL, Science Direct and Cochrane Library were searched from inception to October 2022. Identified records from database literature searches were imported into Covidence^®^. Two independent reviewers screened and extracted the data. The research team collectively created a data charting table/form to standardize data collection. A narrative synthesis was used to summarize the evidence. Six studies (total of 319 participants) that met our eligibility criteria were included in this scoping review. SRFCs can provide high-quality diabetic care, especially for uninsured and economically weaker population. Preliminary evidence further indicate that shared medical appointments and telehealth may facilitate diabetic care especially during times where access to care may be difficult (e.g., COVID lockdown). However, no study included in the review explored or discussed family centred/culturally sensitive interventions. Hence, such interventions should be made part of the curriculum in the future with students in SRFCs exposed to such an approach.

## Introduction

Diabetes is a major cause of morbidity and premature mortality worldwide and now identified as a ‘public health emergency’ and a ‘modern and preventable pandemic’ with a predicted 642 million people to be affected by the year 2040 (1, 2). Unlike type 1 diabetes, which is caused by insulin deficiency due to autoimmune- mediated pancreatic beta- cell failure, type 2 diabetes is characterised by insulin resistance and a degree of beta- cell dysfunction (3). The aetiology of Type Two Diabetes Mellitus (T2DM) comprises of a complex mix of genetic, social, cultural, psychological, political, and economic factors (4, 5). Prevalence rates of type 2 diabetes and obesity have increased in recent decades due to factors such as globalisation and urbanisation, which are accompanied by sedentary behaviour and energy-dense diets (6, 7). Indigenous populations are disproportionately affected by type 2 diabetes and associated complications (4, 8). In Aotearoa/New Zealand for example, 7.2% of Māori (indigenous people) have diabetes compared to 5.1% of Pākehā (New Zealand European). Racism along social determinants of health are root causes of these inequities (8).

Traditionally, the focus of diabetic intervention has been on doctor led primary health strategies. This western medicine-based approach has led to a tendency to measure what can easily be measured (e.g., HbA1c) without much evaluation of team work and transitions of care (9). Also, the current approach does not account much for cultural factors that may act as a barrier for many people (especially indigenous) from accessing care when required (10). The lack of cultural integration means that indigenous and/or socioeconomically disadvantaged people are mere passengers through the system (9–11). Furthermore, for people living in remote/rural places, accessing/commuting to these services may be impractical or may put undue pressure on the family (12–14). Hence, to be effective in terms of prevention and intervention, the current approach may not be sufficient and can be complemented by other approaches including delivery of additional support via relevant curricula innovations and transformation. Placement experience for pre-licensure healthcare student-led clinics or student run free clinics (SRFC) may represent one such strategy whereby pre-licensure healthcare students may make contributions to existing health services, help address service gaps and gain greater insights and hands-on experience in providing services to individuals and families challenge by T2DM.

SRFC’s typically involve pre-licensure students such as student doctors, nurses, physiotherapists, etc. in hands-on practice, particularly within primary health-care settings 1. SRFC’s may involve a single professional group or may be interprofessional in nature. SRFC’s provide an opportunity within the curriculum for teaching population-based medicine, chronic disease assessment and management to medical students (e.g., doctors, nursing, physiotherapy) (1, 15). Further, SRFC’s may also enable students to develop their skills and own practice under close faculty supervision. In turn, this provides an opportunity for the faculty and the student to identify things that are working well and areas that need improvement (4). SRFCs also enable increased access to services, more time for assessments and treatments and more holistic and integrated care for patients.

SRFC’s has been shown to be a useful health delivery model in providing/delivering public health program. A recent systematic review has been shown that SRFCs interventions demonstrated positive impact on patients at risk of developing cardiovascular disease (16, 17). SRFCs have been used to deliver efficient preventive medicine services including HIV testing (9) and falls prevention (18). SRFC may play an important role in providing humanistic care and support to underserved/uninsured and marginalized health communities (12); and those who have difficulty accessing services (19). Although patients have a primary health care provider that oversees and coordinate the quality of care; patients expect more than just a single pointed service or in-coordinated referral. In this context, SRFCs may play an important role in providing this coordinated care to patients with T2DM. Nonetheless, literature about the efficacy of SRFCs specifically addressed in the prevention and management of T2DM to require further development.

Scoping reviews enable to incorporate a range of study designs to comprehensively summarize and synthesize evidence with the aim of informing practice (16). A scoping review was considered appropriate for this review as little is known about the effectiveness of SRFCs in the prevention and management of T2DM.

The aims of this scoping review are to:

- Investigate the opportunity for curriculum enhancement through the role and effectiveness of SRFCs in managing T2DM.
- Establish the barriers and enablers for SRFCs for the management of T2DM diabetes in indigenous population.
- Explore whether a culturally appropriate/sensitive care can be provided through SRFCs in the management of T2DM.

## Methods

This review has been reported in accordance with the preferred reporting items for systematic reviews and meta-analysis extension for scoping review (PRISMA-ScR) checklist (20).

### Eligibility criteria

#### Inclusion criteria

***Participants***: Indigenous Kaumatua (Older adult) with T2DM.

***Intervention***: Any studies (quantitative, qualitative and mixed methods) that investigated mobile health clinic/interventions for people with T2DM will be included in the review.

***Comparison***: Studies will be included with or without a comparison group.

***Outcomes***: Studies will be included if they report any quantifiable outcome and/or qualitative outcome/feedback.

***Setting***: Studies should have taken place only in health care (medicine, nursing, physiotherapy, etc.) setting.

***Limiters***: English language.

#### Exclusion criteria

Studies will be excluded if: (1) they were not conducted in a primary health care setting; (2) the study design is one of the following: secondary research, pilot study, expert opinion, practice guidelines, editorial, letter to the editor, and commentary; (3) non-peer reviewed studies, and (4) non-English studies.

### Information source

The following electronic databases were searched since inception to October 2022: PubMed, CINAHL, Cochrane Library and SCOPUS. Additional search will also be undertaken on protocol registries such as PROSPERO. Furthermore, two reviewers (KK and AY) independently screened the reference list and citations of the included full-text articles for any additional citations.

### Search strategy

The lead investigator developed the initial search strategy which was refined in discussion with an experienced subject librarian. The search strategy was developed to locate studies relevant to three key components of our research question: diabetes mellitus, healthcare inequities and student led clinics. A combination of keywords and MeSH terms such as diabetes OR (Health Services, Indigenous) OR (Healthcare Disparities) OR (Medically Underserved Area) OR (Student Run Clinic) were used. The search strategy was developed and adapted for various databases. An example of this process has been provided in Appendix 1.

### Study records

#### Data management

Identified records from database literature searches were imported into Covidence^®^ (17), an online data management software. Automatic removal of duplicates in Covidence was followed by a two-stage screening of unique studies by two sets of independent reviewers (KK and AY).

#### Study selection

Titles and abstracts of the retrieved articles were screened independently by two reviewers (KK and AY) for relevance after removing the duplicates. Full-text articles that did not meet the inclusion criteria were excluded. Any disagreements that arose between reviewers at any stage of the selection process were resolved through discussion; if no agreement could be reached, a third reviewer (SB) was available to be consulted.

#### Data collection process

The research team collectively created a data charting table/form to standardise data collection. Two independent reviewers (KK and AY) appraised the extracted data, with the opportunity to consult a third reviewer (MH) in case of disagreement. Data that extracted from each study include in whole or combination study’s aim; study design; participant demographics, service provided, outcome measures, and findings.

#### Summarising the data

A narrative synthesis was used to summarise the data. The data were summarised under the following key concepts which were considered important: (1) intervention/care provided; (2) role of students; (3) outcome measures used; (4) Quality of care of diabetes in SRFC; (5) patient satisfaction; and (6) type of consultation.

#### Quality assessment (including risk of bias)

This was not undertaken as this was not considered mandatory for a scoping review.

## Results

**Figure 1:**
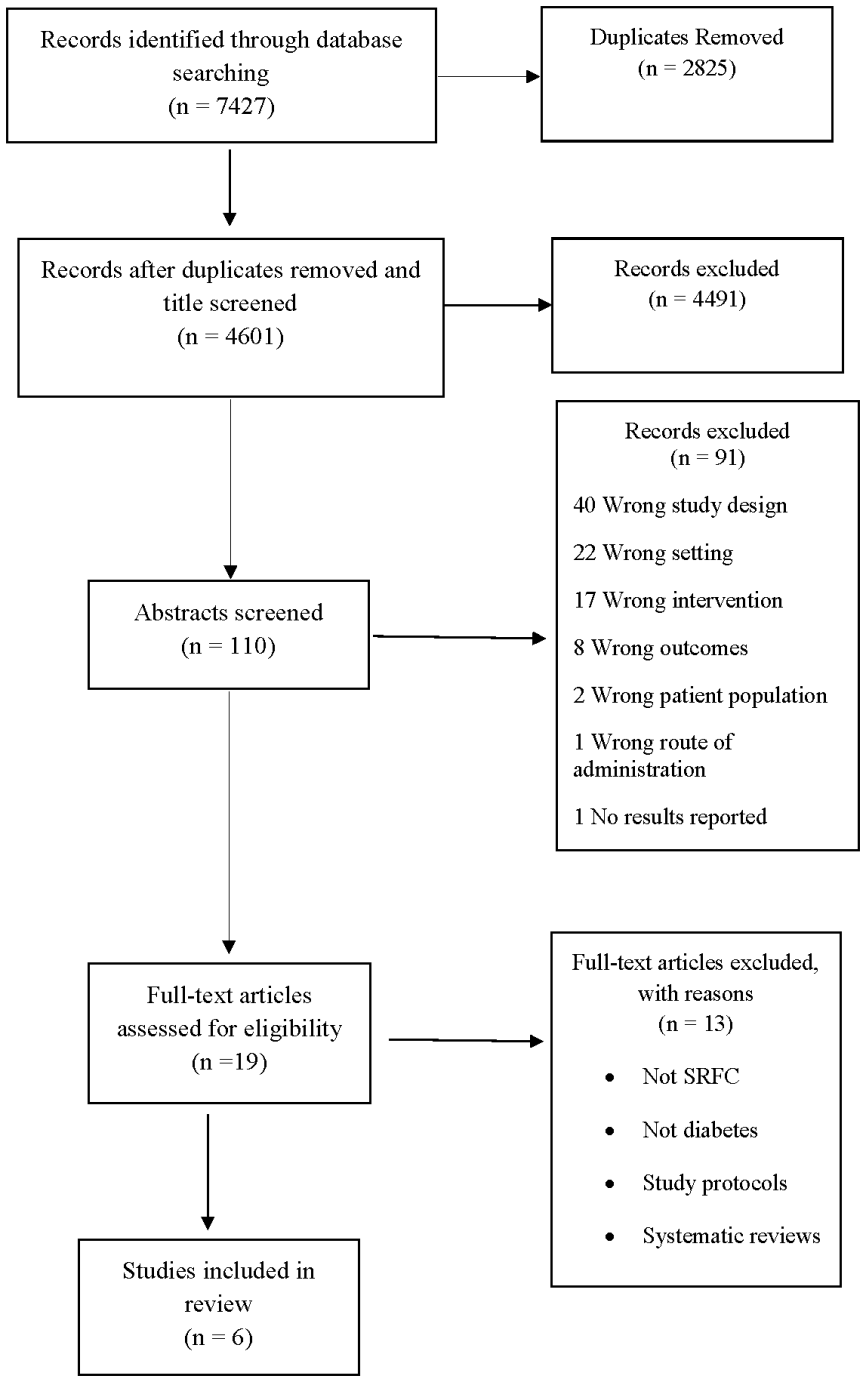
PRISMA flow diagram of included studies.

The electronic search yielded a total of 7,427 articles. Following the removal of duplicates, 4,601 articles were retained for further screening. After title, abstract, and full-text screening, only 6 studies (21–26) met our criteria and were included in our review (refer Figure 1).

### Characteristics of included studies

Characteristics of the 6 included studies are presented in Table 1. The number of participants with T2DM ranged between 8 to 182 and included a total of 319 patients. All studies included both male and female participants. The ethnicity of participants varied including African, Asian, Latino, Hispanic, Pacific Islanders and White. All the six studies were undertaken in the United States of America.

**Table 1:** Characteristics of included studies.

Study ID/Country/Name of program	Study objectives	Study design	Participant demographics	Service provided/duration	Outcome measures/duration	Main findings
Gorrindo 2014; United States of America; Shade Tree Clinic Patient Health Education (PHE) program.	To examine the clinical impact of a medical student health educator program for diabetic patients	Retrospective study design	Total: 45	Free medical care, medications, laboratory services, immunizations, social services, and disease management.	Mean A1c 9.6	A medical student health educator program at an SRFC can provide high-quality diabetes care and facilitate clinical improvement 1 year after enrolment, despite inherent difficulties in caring for underserved patients.
			Ethnicity:	Educational activities include student-led preclinic â€œchalk talksâ€ (small-group discussions of clinical topics relevant to patients scheduled in the clinic), faculty-led postclinic â€œwrap-upâ€ discussions that afford students an opportunity to share interesting or particularly educational cases they saw in the clinic that day, weekly laboratory review sessions, quarterly case presentation series, and annual clinical skills workshops.		
			Hispanic 15/45 (33.3%)			
			Non-Hispanic white 13/45 (28.9%)			
			Non-Hispanic black 16/45 (35.6%)			
			Non-Hispanic other 1/45 (2.2%)			
			Age:	Duration: 1 year		
			48.7 (10.3)			
			Gender:			
			Male (37.8%)			
			Female (62.2%)			
Felder-Heim 2020; United States of America; DAWN (Dedicated to Auroraâ€^™^s Wellness and Needs).	To understand DAWNâ€^™^s ability to achieve quality-of-care performance standards for diabetes and hypertension similar to other safety-net providers, and to identify quality improvement targets that may lead to improved chronic disease management.	Retrospective chart review	Total: 30	HbA1c screen, nephropathy screen (or ACE-inhibitor prescription), retinopathy screen, lipid panel, and prescription.	HbA1c, neuropathic symptoms, retinopathy screen and lipid levels.	SRFC may have a role in safety net health care system.
			Ethnicity:			
			NA			
			Indigenous 6 (75%)			
			Non-Hispanic White			
			2 (25%)			
			Age:			
			19–44 7/30 (23.3%)			
			45–64 16/30 (53.3%)			
			65–74 5/30 (16.7%)			
			75–84 2/30 (6.7%)			
			Gender:			
			Male (60%)			
			Female (40%)			
Kahkoska 2018; United States of America; Student Run Free Clinics (SRFC).	The objective was to increase patient engagement and improve health outcomes in this underserved patient population by transitioning from the traditional clinical model to the patient-driven SMA model	Prospective evaluation study	Total: 8	Teams of transdisciplinary trainees work together to perform triage, medication reconciliation, brief history, and physical exam, after which patients participate in the shared medical appointments (SMA). The endocrinologist evaluates SMA patients individually during and after the visit	HbA1c	SMA may help address health disparities and increase the quality of free diabetes care.
			Ethnicity:			
			Indigenous 6 (75%)			
			Non-Hispanic White			
			2 (25%)	Duration: 2 years		
			Gender:			
			Male (75%)			
			Female (25%)			
Schroeder 2020; United States of America; Community Care Free Medical Clinic (CCFMC)	The primary objective of this quality improvement study was to assess patient satisfaction with diabetes care at an SRFC.	Survey study design	Total: 25	Duration: 7 weeks.	The Shade Tree Patient Satisfaction Survey, Diabetes Treatment Satisfaction Questionnaire, and Diabetes Self-Management Questionnaire	The survey helped identify key areas in which the diabetes care provided at the SRFC could be improved. These areas included education about diabetes in general, as well as in understanding treatment, self-monitoring, and healthy eating and exercise options.
			Ethnicity: White (17); Hispanic (3)			
			Black African/American (3); Native American (1); Asian/pacific Islander (1)			
			Age:			
	In addition to satisfaction of overall diabetes care, the study focused on satisfaction of self-management of diabetes, nutrition, and exercise.		56 (Range: 25–67)			
	Secondary objectives included evaluating satisfaction between ages, sex, length of diabetes diagnosis, and time attending the CCFMC.		Gender:			
			Male (15)			
			Female (10)			
Simon 2022; United States of America; Student Run Free Clinics (SRFC).	The aim of this study is to evaluate the impact of the pandemic on the management of chronic disease, specifically diabetes.	Retrospective study design	Total: 29	Eye exam, chronic kidney disease monitoring, Hb A1c Value, BP, influenza vaccination and prescribed statin therapy.	Eye exam, chronic kidney disease monitoring, Hb A1c Value, BP, influenza vaccination and prescribed statin therapy.	Diabetes care using telehealth in a SRFC may be an acceptable alternative model when face-to-face visits are not feasible.
			Ethnicity:			
			Hispanic			
			Non-Hispanic			
			Other			
			Age:			
			Hispanic			
			Non-Hispanic			
			Other			
			Gender:			
			Male (16)			
			Female (13)			
Smith 2014; United States of America; University of California San Diego (UCSD) Student Run Free Clinic (SRFC).	To determine if the quality of care of diabetic patients at a Student-Run Free Clinic Project (SRFCP) meets the standard of care, is comparable with other published outcomes, and whether pertinent diabetic clinical indicators improve over time	Retrospective chart review	Total: 182	Screening tests (process measures) was blood pressure (BP) 100%, HbA1c 99.5%, creatinine 99.5%, LDL 93%, HDL and triglycerides 88%, microalbumin/creatinine ratio 80%, and ophthalmology exam 32%.	Blood pressure (BP)	Diabetic patients at UCSD SRFCP reached goals for both process measures and intermediate outcomes at rates that meet or exceed published outcomes of insured and uninsured diabetics on nearly all measures, with the exception of ophthalmology screening.
			Ethnicity:		HbA1c	
			Latino (75%)			
			Caucasian (15%)		Creatinine	
			Asian (4%)			
			African American		LDL	
			(3%)			
			Other (3%)	Duration: 1 year	HDL	
			Age:			
			53 (11.5)		Triglycerides 88%, Microalbumin/creatinine ratio	
			Gender:			
			Male (41%)		Ophthalmology exam 32%.	
			Female (59%)			

### Intervention/care provided

The nature of intervention/care provided varied across the studies and included screening tests (including ophthalmology exam), immunizations, medical care, medications, laboratory services, social services, disease management, exercise and patient education. The duration of care also varied among studies and ranged between 7 weeks to 2 years.

### Role of students

Although all studies had students on placement and/or providing care, only two studies reported on the role of students and the nature of the placements. In the study by Gorrindo et al. (22), pre-clinical and clinical students had a twice-weekly clinic sessions under the supervision of faculty providers. Other educational activities included student-led preclinic “chalk talks” and faculty-led postclinic “wrap-up” discussions. In the study by Kahkoska et al. (23), teams of transdisciplinary trainees work together to perform triage, medication reconciliation, brief history, and physical exam.

### Outcome measures

The most common outcome measures used across the studies included physiological measures such as BP, HbA1c, lipid levels, eye exam, retinopathy, and neuropathic screen. Few studies also used outcome measures such as American Diabetes Association (ADA) process and outcome measure benchmarks to track success of the care provided by SRFC. Patient satisfaction was also measured (24) using tools such as The Shade Tree Patient Satisfaction Survey, Diabetes Treatment Satisfaction Questionnaire, and Diabetes Self-Management Questionnaire.

#### Quality of care of diabetes in SRFC

Three included studies investigated the quality of care of diabetic patients at a SRFCs and whether the quality of care at SRFCs are comparable with other published outcomes. Gorrindo et al. (22) examined the clinical impact of a medical student health educator program for diabetic patients at an SRFC. This involved retrospectively reviewing the electronic medical records of diabetic patients for 3 years. They compared clinical outcomes at initial presentation to the clinic and 12 months later and analyzed the relationship between the number of patient–student interactions (touchpoints) and change in haemoglobin A1c values. Further, the quality of care provided was compared to best-practice benchmarks (process and outcomes measures). The mean haemoglobin A1c values improved significantly. The authors concluded that a SRFC can provide high quality diabetes care and facilitate clinical improvement 1 year after enrolment. Smith et al. (26) conducted a retrospective review of diabetic patients at three SRFCs (*n* = 182) and compared the quality of care with published outcomes. The study reported that diabetic patients at these SRFCs reached goals for both process measures and intermediate outcomes at rates that meet or exceed published outcomes of insured and uninsured diabetics on nearly all measures. Felder-Heim and Mader (21) investigated DAWN (Dedicated to Aurora’s Wellness and Needs) SRFC’s ability to achieve quality-of-care performance standards for diabetes and hypertension similar to other safety-net providers. A mixed-methods evaluation of diabetes and hypertension management was conducted for patients. Retrospective chart review assessed whether patients received recommended screening tests (process outcomes) and achieved disease control (short-term outcomes). In-depth case studies of randomly selected individuals with good and poor disease control identified targets for quality improvement through nominal group technique. The outcomes were compared to local health centres. SRFC may have a role in safety net health care system.

#### Patient satisfaction

Schroeder and Hickey (24) used survey methodology to assess patient satisfaction with diabetes care at a SRFC in order to assist in identifying areas of improvement. Established patients who were aged 18 years or older and diagnosed with diabetes, were invited to complete the survey. The majority of patients (88%) were satisfied with their diabetes care at the SRFC. Sub analyses demonstrated significant differences when comparing sex, age, and length of diabetes diagnosis. Areas of improvement were identified including education about diabetes in general, as well as in understanding treatment, self-monitoring, and healthy eating and exercise options.

#### Type of consultation

Two studies investigated the effects of type of consultation (face to face vs. telehealth and shared medical appointment) on quality of care of diabetes in SRFCs. Simon et al. (25) evaluated the impact of the pandemic on the management of chronic disease, specifically diabetes. Patients with diabetes who received care continuously throughout the pre-pandemic (face-to-face) and pandemic (telehealth) study periods at a SRFC were evaluated. The progress was evaluated on six quality measures including annual eye exams, blood pressure, hemoglobin A1c, chronic kidney disease monitoring, fu vaccination, and statin therapy. The study demonstrated that diabetes care using telehealth in a SRFC may be an acceptable alternative model when face-to-face visits are not feasible. Kahkoska et al. (23) explored whether shared medical appointments (SMA) improve outcomes in type 2 diabetes. SMA groups comprised of transdisciplinary trainees working together to perform triage, medication reconciliation, brief history, and physical exam, after which patients participate in the SMA. The endocrinologist evaluated SMA patients individually during and after the visit. The study reported that SMA increased clinic efficiency and offered an opportunity to integrate transdisciplinary trainees.

## Discussion

### Summary of findings

This scoping review aimed to investigate the role and effectiveness of student led clinics in managing T2DM. A key finding of our review was that SRFCs can provide high-quality diabetic care, especially for uninsured and economically weaker population. These improvements are observed in both physiological outcome measures and logistical processes. Preliminary evidence further indicate that shared medical appointments and telehealth may facilitate diabetic care especially during times where access to care may be difficult (e.g., COVID-19 lockdown).

Our review found strong evidence that SRFCs are effective in the management of T2DM (21–23). This is not only consistent with published literature on the management of DM but also other chronic medical conditions, such as hypertension and smoking cessation (18, 27–29). Hence it can be argued that SRFCs can be used as conduits for effective DM care. Interestingly, the outcomes from these SRFCs (where students are supervised by clinicians) compared well with that of normal medical care provided by health professionals (26). Taken together, our findings and the existing literature, it is evident that medical students can design and implement good management plans that may meet the standards of care for patients with T2DM.

The quality of care provided at SRFCs has been a matter of debate. However, our review found that patients were satisfied with the care provided by students (24). This is in agreement with previous findings that showed that the quality of care provided at SRFCs are comparable or better than other providers. Further, shared medical appointments that involved transdisciplinary teams not only provided quality of care but also expedited patient intake (23). Interestingly, the SRFC care provided via telehealth during the COVID pandemic was also found to be effective and resulted in patient satisfaction (25). Collectively, these findings point to a bigger role of SRFCs in the management of T2DM.

On the other hand, however, our review identified a number of aspects of SRFC that can be improved including consistent patient education, monitoring and tracking of patient’s diet and physical activity (24). A key strategy that may need to be incorporated as part of SRFC would be ‘goal setting’ with patients where healthy eating and counselling are part of goal setting (30). In this context, a SRFC that promotes inter-professional education may be important to expose students to a multi-dimensional approach to DM. Such an approach may not only benefit the students from variety of clinical experiences but also would facilitate students’ experience in addressing this major public health issues and in understanding of other professions and prepare them for future practice (for example, SMA) (23, 31). Hence, it seems timely strengthen the public health focus for undergraduate healthcare students and strengthening inter-professional knowledge and insights as part of undergraduate health curriculum.

All studies in the current review included patients from disadvantaged communities, especially of Hispanic and African ethnicities. While all studies reported improvements in metabolic measures, it is unclear whether any culturally appropriate/safe interventions were provided. Traditionally, the focus of diabetic intervention has been on doctor and nurse-led primary health strategies involving physical activity and nutrition components that are effective at preventing diabetes and cardiovascular disease along with reducing weight (9, 32). However, evidence-based interventions may not be effective in indigenous communities without adapting the intervention to fit the target community (9, 19, 33). Family-centred interventions may play an important role in this context (34). This may include supporting healthy family behaviours; promoting community connectedness; improving access and culturally supportive care. For example, many indigenous older adult live in family home (11) with their families and do not necessarily cook for themselves alone and may not east nutritious and/or the right type of food for T2DM. Hence, it may be important for SRFCs to understand the kind of foods people from minority ethnic groups are accustomed to and prepare educational resources based on that information. Preliminary evidence suggests that such an approach may improve diet quality, hypertension and BMI (35). Further, promoting a cultural, spiritual and community connectedness is also an important strategy to facilitate a holistic management for T2DM (11, 34). This includes identifying, training and employing an indigenous health care workforce and providing health care delivery information in native languages (33, 34). However, no study included in the review explored or discussed family centred interventions. Hence, family-centred interventions should be made part of the curriculum with students in SRFCs exposed to such an approach.

### Limitations

The review is not without its limitations. Only a small number (six) studies met our inclusion criteria. Further, the included studies were heterogeneous which may limit the confidence in our findings. However, we carried out an exhaustive search and maximised opportunity to include studies. Hence, the small number of studies may point to an emerging field and/or need for more research in this area. All the studies included in the review were done in the United States of America. Therefore, the generalizability of the findings to other countries, setting and health systems can be limited. Secondly, the nature of training and the role of students was varied and heterogeneous across studies. For example, only one study had reported the educational activities provided to students. This may seriously limit our ability to make any recommendations about the educational content for students in the SRFC. All studies included people from disadvantaged communities who were mainly of Hispanic or African ethnicity. Future studies should investigate the effectiveness of SRFC in the management of T2DM in other indigenous communities. Family centred and community centred health care models may be timely in preventing the pandemic of T2DM for which SRFCs may play a crucial role. Hence, future programs should consider incorporating such health care models as part of their curriculum.

### Recommendations

Based on our scoping review findings, the following recommendations are made:

- SRFC have an important role in managing and preventing the T2DM pandemic. Hence, the curriculum for health care professionals must be reviewed to include greater focus of this major public health crisis.
- The curriculum for health care professionals must include holistic management strategy of T2DM and not just metabolic outcome measures.
- Cultural aspects/understanding has been shown to be a barrier for managing T2DM. Hence students must be exposed to family/community centred health care models that promote cultural understanding, particularly for indigenous and vulnerable population.

## Conclusion

The findings from the current review suggests that SRFC may play an important role in complimenting core services and expanding support to patients with T2DM. Our review further found that patients were satisfied with the care provided by students. However, the cultural aspects of SRFC are an area of future research.

## Author contributions

KK, YA-R, MH, TT, and SB provided substantial contributions to this work and accept accountability for the finished product. KK conceived the scoping review. YA-R developed the search strategy. KK, YA-R, and SB participated in the collection of data and analysis including COVIDENCE screening. MH and TT provided critical inputs. All authors contributed to the article and approved the submitted version.

## Funding

This project is supported by a Health Research Council (NZ) Grant ref: 21–1070.

## Conflict of interest

TT was employed by Tu Tonu Rehabilitation Ltd.

The remaining authors declare that the research was conducted in the absence of any commercial or financial relationships that could be construed as a potential conflict of interest.

## Publisher’s note

All claims expressed in this article are solely those of the authors and do not necessarily represent those of their affiliated organizations, or those of the publisher, the editors and the reviewers. Any product that may be evaluated in this article, or claim that may be made by its manufacturer, is not guaranteed or endorsed by the publisher.
