# Supplementary material for: Exploring the option of student-run free health clinics to support people living with type 2 diabetes mellitus: a scoping review
Source: Front Public Health. 2023 Jul 18;11:1128617. doi: 10.3389/fpubh.2023.1128617 (PMC10392832; doi:10.3389/fpubh.2023.1128617)
Supplement: Supplementary file 1 [file Data_Sheet_1.PDF]

Search strategy

Databases:

CINAHL, Cochrane Library, PubMed, SCOPUS

Subject headings and Keywords

Concepts	MeSH	CINHAL Subject Headings	Keywords
Diabetes Mellitus	(MH "Diabetes Mellitus, Type 2+")	(MM "Diabetes Mellitus, Type 2") OR<br>(MM “diabetic Patients”)	Diabetes OR<br>diabetic
Healthcare inequalities	(MM "Health Services for Persons with Disabilities") OR<br>(MM "Health Services, Indigenous") OR<br>(MM "Health Services for the Aged") OR<br>(MM "Healthcare Disparities") OR<br>(MM "Medically Underserved Area") OR<br>(MM "Mobile Health Units") OR<br>(MM "Student Run Clinic")	(MM "Health Services for Persons with Disabilities") OR<br>(MM "Health Services for the Indigent") OR<br>(MM "Health Services, Indigenous") OR<br>(MM "Health Services for Older Persons") OR<br>(MM "Healthcare Disparities") OR<br>(MM “medically underserved Area”)<br>(MM "Mobile Health Units")	first-nations OR<br>indigenous OR<br>aboriginal OR<br>kaumatua OR<br>kui OR<br>kuia OR<br>marae OR<br>maori OR<br>Mobile-health-clinic* OR<br>mobile-clinic* OR<br>mobile-medic* OR<br>outreach-clinic* OR<br>outreach-service* OR<br>community-outreach OR<br>student-run OR<br>student-led OR<br>(Health* N3 equity) OR<br>(Health* N3 inequalit*)

CINHAL

(n=1473)

((MM "Health Services for Persons with Disabilities") OR (MM "Health Services for the Indigent") OR (MM "Health Services, Indigenous") OR (MM "Health Services for Older Persons") OR (MM "Healthcare Disparities") OR (MM “medically underserved Area”) OR (MM "Mobile Health Units") OR AB ((Health* N3 inequalit*) OR (Health* N3 equity) OR first-nations OR indigenous OR aboriginal OR kaumatua OR kui OR kuia OR marae OR maori OR mobile-health-clinic* OR mobile-clinic* OR mobile-medic* OR outreach-clinic* OR outreach-service* OR community-outreach OR student-run OR student-led)) AND ((MM "Diabetes Mellitus, Type 2") OR (MM “diabetic Patients”) OR AB (diabetes OR diabetic))

Cochrane

(Trials=269, Editorial=3)

Title-Abstract-Keyword (((Health* NEAR/3 equity) OR (Health* NEAR/3 inequalit*) OR first-nations OR indigenous OR aboriginal OR kaumatua OR kui OR kuia OR marae OR maori OR mobile-health-clinic* OR mobile-clinic* OR mobile-medic* OR outreach-clinic* OR outreach-service* OR community-outreach OR student-run OR student-led) AND (diabetes OR diabetic))

Limiters: 2011-2022

PubMed

(n=2902)

(((Health* n3 equity[Title/Abstract]) OR (Health* n3 inequalit*[Title/Abstract]) OR "first-nations"[Title/Abstract] OR "indigenous"[Title/Abstract] OR "aboriginal"[Title/Abstract] OR "kaumatua"[Title/Abstract] OR "kui"[Title/Abstract] OR "kuia"[Title/Abstract] OR "marae"[Title/Abstract] OR "maori"[Title/Abstract] OR "mobile health clinic*"[Title/Abstract] OR "mobile clinic*"[Title/Abstract] OR "mobile medic*"[Title/Abstract] OR "outreach clinic*"[Title/Abstract] OR "outreach service*"[Title/Abstract] OR "community-outreach"[Title/Abstract]) OR ("Health Services for Persons with Disabilities"[All Fields] OR "health services indigenous"[All Fields] OR "Health Services for the Aged"[All Fields] OR "Healthcare Disparities"[All Fields] OR "Medically Underserved Area"[All Fields] OR "Mobile Health Units"[MeSH Terms])) AND (("diabetes"[Title/Abstract] OR "diabetic"[Title/Abstract]) OR ("diabetes mellitus, type 2"[MeSH Major Topic]))

Limiters: 2011-2022 and English

Scopus

(n=3640)

(**TITLE-ABS-KEY**((Health* W/3 equity) OR (Health* W/3 inequalit*) OR first-nations OR indigenous OR aboriginal OR kaumatua OR kui OR kuia OR marae OR maori OR mobile-health-clinic* OR mobile-clinic* OR mobile-medic* OR outreach-clinic* OR outreach-service* OR community-outreach OR student-run OR student-led) AND **TITLE-ABS-KEY**(diabetes OR diabetic))

Limiters: 2011-2022 and English
