# Supplementary material for: Exploring the option of student-run free health clinics to support people living with type 2 diabetes mellitus: a scoping review
Source: Front Public Health. 2023 Jul 18;11:1128617. doi: 10.3389/fpubh.2023.1128617 (PMC10392832; doi:10.3389/fpubh.2023.1128617)
Supplement: Supplementary file 2 [file Data_Sheet_2.PDF]

Study ID/Country/Name of program	Study Objectives	Study Design	Participant demographics	Service Provided/Duration	Outcome measures/duration	Main Findings
Smith 2014; United States of America; University of California San Diego (UCSD) Student Run Free Clinic (SRFC).	To determine if the quality of care of diabetic patients at a Student-Run Free Clinic Project (SRFCP) meets the standard of care, is comparable with other published outcomes, and whether pertinent diabetic clinical indicators improve over time	Retrospective chart review	Total: 182<br>Ethnicity:<br>Latino (75%)<br>Caucasian (15%)<br>Asian (4%)<br>African American (3%)<br>Other (3%)<br>Age:<br>53 (11.5)<br>Gender:<br>Male (41%)<br>Female (59%)	screening tests (process measures) was blood pressure (BP) 100%, HbA1c 99.5%, creatinine 99.5%, LDL 93%, HDL and triglycerides 88%, microalbumin/creatinine ratio 80%, and ophthalmology exam 32%.<br><br>Duration: 1 year	Blood pressure (BP)<br>HbA1c<br>Creatinine<br>LDL<br>HDL<br>Triglycerides 88%,<br>Microalbumin/creatinine ratio<br>Ophthalmology exam 32%.	Diabetic patients at UCSD SRFCP reached goals for both process measures and intermediate outcomes at rates that meet or exceed published outcomes of insured and uninsured diabetics on nearly all measures, with the exception of ophthalmology screening.
Gorrindo 2014; United States of America; Shade Tree Clinic Patient Health Education (PHE) program.	To examine the clinical impact of a medical student health educator program for diabetic patients	Retrospective study design	Total: 45<br>Ethnicity:<br>Hispanic 15/45 (33.3%)<br>Non-Hispanic white 13/45 (28.9%)	free medical care, medications, laboratory services, immunizations, social services, and disease management.	mean A1c 9.6	A medical student health educator program at an SRFC can provide high-quality diabetes care and facilitate clinical improvement one

			Non-Hispanic black 16/45 (35.6%)<br>Non-Hispanic other 1/45 (2.2%)<br>Age:<br>48.7 (10.3)<br>Gender:<br>Male (37.8%)<br>Female (62.2%)	Educational activities include student-led preclinic â€œchalk talksâ€ (small-group discussions of clinical topics relevant to patients scheduled in the clinic), faculty-led postclinic â€œwrap-upâ€ discussions that afford students an opportunity to share interesting or particularly educational cases they saw in the clinic that day, weekly laboratory review sessions, quarterly case presentation series, and annual clinical skills workshops.<br>Duration: 1 year		year after enrolment, despite inherent difficulties in caring for underserved patients.
Schroeder 2020; United States of America; Community Care Free Medical Clinic (CCFMC)	The primary objective of this quality improvement study was to assess patient satisfaction with diabetes care at an SRFC.	Survey study design	Total: 25<br>Ethnicity:<br>White (17);<br>Hispanic (3);<br>Black African/American (3); Native American (1);<br>Asian/pacific Islander (1)	Duration: 7 weeks.	The Shade Tree Patient Satisfaction Survey, Diabetes Treatment Satisfaction Questionnaire, and Diabetes Self-Management Questionnaire	The survey helped identify key areas in which the diabetes care provided at the SRFC could be improved. These areas included education about diabetes in general, as well as in

	In addition to satisfaction of overall diabetes care, the study focused on satisfaction of self-management of diabetes, nutrition, and exercise. Secondary objectives included evaluating satisfaction between ages, sex, length of diabetes diagnosis, and time attending the CCFMC.		Age:<br>56 (Range: 25-67)<br>Gender:<br>Male (15)<br>Female (10)			understanding treatment, self-monitoring, and healthy eating and exercise options.
Simon 2022; United States of America; Student Run Free Clinics (SRFC).	The aim of this study is to evaluate the impact of the pandemic on the management of chronic disease, specifically diabetes.	Retrospective study design	Total: 29<br>Ethnicity:<br>Hispanic<br>Non-Hispanic<br>Other<br>Age:<br>Hispanic<br>Non-Hispanic<br>Other<br>Gender:<br>Male (16)	Eye exam, chronic kidney disease monitoring, Hb A1c Value, BP, influenza vaccination and prescribed statin therapy.	Eye exam, chronic kidney disease monitoring, Hb A1c Value, BP, influenza vaccination and prescribed statin therapy.	Diabetes care using telehealth in a SRFC may be an acceptable alternative model when face-to-face visits are not feasible.

			Female (13)			
Kahkoska 2018; United States of America; Student Run Free Clinics (SRFC).	The objective was to increase patient engagement and improve health outcomes in this underserved patient population by transitioning from the traditional clinical model to the patient-driven SMA model	Prospective evaluation study	Total: 8<br>Ethnicity:<br>Indigenous 6 (75%)<br>Non-Hispanic White 2 (25%)<br>Gender:<br>Male (75%)<br>Female (25%)	Teams of transdisciplinary trainees work together to perform triage, medication reconciliation, brief history, and physical exam, after which patients participate in the shared medical appointments (SMA). The endocrinologist evaluates SMA patients individually during and after the visit<br>Duration: 2 years	HbA1c	SMA may help address health disparities and increase the quality of free diabetes care.
Felder-Heim 2020; United States of America; DAWN (Dedicated to Auroraâ€™s Wellness and Needs).	To understand DAWNâ€™s ability to achieve quality-of-care performance standards for diabetes and hypertension similar to other safety-net providers, and to identify quality improvement targets that may lead to improved	Retrospective chart review	Total: 30<br>Ethnicity:<br>NA<br>Indigenous 6 (75%)<br>Non-Hispanic White 2 (25%)<br>Age:<br>19-44 7/30 (23.3%)<br>45-64 16/30 (53.3%)<br>65-74 5/30 (16.7%)	HbA1c screen, nephropathy screen (or ACE-inhibitor prescription), retinopathy screen, lipid panel, and prescription.	HbA1c, Neuropathic Symptoms, retinopathy screen and lipid levels.	SRFC may have a role in safety net health care system.

	chronic disease management.		75-84 2/30 (6.7%)<br>Gender:<br>Male (60%)<br>Female (40%)			
